# Supplementary material for: Spatial and semantic memory reorganize a hippocampal long-axis gradient
Source: bioRxiv. 2025 Oct 15:2025.10.15.682651. Preprint. [Version 1] doi: 10.1101/2025.10.15.682651 (PMC12632773; doi:10.1101/2025.10.15.682651)
Supplement: 5 [file NIHPP2025.10.15.682651v1-supplement-5.pdf]

bioRxiv preprint doi: https://doi.org/10.1101/2025.10.15.682651; this version posted October 15, 2025. The copyright holder for this preprint (which was not certified by peer review) is the author/funder, who has granted bioRxiv a license to display the preprint in perpetuity. It is made available under aCC-BY 4.0 International license.

Supplementary Information

Supplementary Methods

Anatomical data preprocessing

Anatomical data were preprocessed using *fMRIPrep* 23.1.4 [98]. A total of 2 T1-weighted (T1w) images were found per subject within the input BIDS dataset. All of them were corrected for intensity non-uniformity (INU) with `N4BiasFieldCorrection` [99], distributed with ANTs [100]. The T1w-reference was then skull-stripped with a *Nipype* implementation of the `antsBrainExtraction.sh` workflow (from ANTs), using OASIS30ANTs as target template. Brain tissue segmentation of cerebrospinal fluid (CSF), white-matter (WM) and gray-matter (GM) was performed on the brain-extracted T1w using `fast` (FSL version 6.0.7.7, [101]). An anatomical T1w-reference map was computed after registration of 2 T1w images (after INU-correction) using `mri_robust_template` (FreeSurfer version 7.3.2, [102]). Brain surfaces were reconstructed using `recon-all` (FreeSurfer version 7.3.2, [103]), and the brain mask estimated previously was refined with a custom variation of the method to reconcile ANTs-derived and FreeSurfer-derived segmentations of the cortical gray-matter of Mindboggle [104]. Volume-based spatial normalization to one standard space (MNI152NLin2009cAsym) was performed through nonlinear registration with `antsRegistration` (ANTs, version 2.5.1), using brain-extracted versions of both T1w reference and the T1w template. The following template was selected for spatial normalization and accessed with *TemplateFlow* (version 23.0.0, [105]): *ICBM 152 Nonlinear Asymmetrical template version 2009c* [106].

Functional data preprocessing

For each of the BOLD runs found per subject (up to 16 across sessions), the following preprocessing was performed. First, a reference volume and its skull-stripped version were generated using a custom methodology of *fMRIPrep*. Head-motion parameters with respect to the BOLD reference (transformation matrices, and six corresponding rotation and translation parameters) are estimated before any spatiotemporal filtering using `mcflirt` (FSL, [107]). BOLD runs were slice-time corrected to 1.0s (0.5 of slice acquisition range 0s-2.0s) using `3dTshift` from AFNI [108]. The BOLD time-series (including slice-timing correction when applied) were resampled onto their original, native space by applying the transforms to correct for head motion. These resampled BOLD time-series will be referred to as preprocessed BOLD in original space, or just preprocessed BOLD. The BOLD reference was then co-registered to the T1w reference using `bbregister` (FreeSurfer) which implements boundary-based registration [109]. Co-registration was configured with six degrees of freedom. Several confounding time-series were calculated based on the preprocessed BOLD: framewise displacement (FD), DVARS and three region-wise global signals. FD was computed using two formulations following Power (absolute sum of relative motions, [110]) and Jenkinson (relative root mean square displacement between affines, [107]). FD and DVARS are calculated for each functional run, both using their implementations in *Nipype* following the definitions Power et al. [110]. The three global signals are extracted within the CSF, the WM, and the whole-brain masks. Additionally, a set of physiological regressors were extracted to allow for component-based noise correction (*CompCor*, [111]). Principal components are estimated after high-pass filtering the preprocessed BOLD time-series (using a discrete cosine filter with 128s cut-off) for the two *CompCor* variants: temporal (tCompCor) and anatomical (aCompCor). tCompCor components are then calculated from the top 2% variable voxels within the brain mask. For aCompCor, three probabilistic masks (CSF, WM and combined CSF+WM) are generated in anatomical space. The implementation differs from that of Behzadi et al. in that instead of eroding the masks by 2 pixels on BOLD space, a mask of pixels that likely contain a volume fraction of GM is subtracted from the aCompCor masks. This mask is obtained by dilating a GM mask extracted from the FreeSurfer's *aseg* segmentation, and it ensures components are not extracted from voxels containing a minimal fraction of GM. Finally, these masks are resampled into BOLD space and binarized by thresholding at 0.99 (as in the original implementation). Components are also calculated separately within the WM and CSF masks. For each CompCor decomposition, the k components with the largest singular values are retained, such that the retained components' time series are sufficient to explain 50 percent of variance across the nuisance mask (CSF, WM, combined, or temporal). The remaining components are dropped from consideration. The head-motion estimates calculated in the correction step were also placed

bioRxiv preprint doi: https://doi.org/10.1101/2025.10.15.682651; this version posted October 15, 2025. The copyright holder for this preprint (which was not certified by peer review) is the author/funder, who has granted bioRxiv a license to display the preprint in perpetuity. It is made available under aCC-BY 4.0 International license.

within the corresponding confounds file. The confound time series derived from head motion estimates and global signals were expanded with the inclusion of temporal derivatives and quadratic terms for each [112]. Frames that exceeded a threshold of 0.5 mm FD or 1.5 standardized DVARS were annotated as motion outliers. Additional nuisance timeseries are calculated by means of principal components analysis of the signal found within a thin band (*crown*) of voxels around the edge of the brain, as proposed by Patriat et al. [113]. The BOLD time-series were resampled into standard space, generating a preprocessed BOLD run in MNI152NLin2009cAsym space. First, a reference volume and its skull-stripped version were generated using a custom methodology of *fMRIPrep*. All resamplings can be performed with a single interpolation step by composing all the pertinent transformations (i.e. head-motion transform matrices, susceptibility distortion correction when available, and co-registrations to anatomical and output spaces). Gridded (volumetric) resamplings were performed using `antsApplyTransforms` (ANTs), configured with Lanczos interpolation to minimize the smoothing effects of other kernels [114]. Non-gridded (surface) resamplings were performed using `mri_vol2surf` (FreeSurfer).

bioRxiv preprint doi: https://doi.org/10.1101/2025.10.15.682651; this version posted October 15, 2025. The copyright holder for this preprint (which was not certified by peer review) is the author/funder, who has granted bioRxiv a license to display the preprint in perpetuity. It is made available under aCC-BY 4.0 International license.

Cluster Type	x	y	z	Size (mm^3)	BS	LS	OS	LS vs. BS	OS vs. BS	OS vs. LS
**Object**										
	-28.2	-15.5	-19.1	95	-0.19	0.34	**3.13**	0.31	1.24	1.67
**Location**										
	28.6	-35.0	-4.7	280	0.11	**3.37**	1.69	1.41	0.91	-0.81
**Both**										
	-33.1	-23.6	-13.7	304	**3.57**	-0.19	0.14	**-3.12**	**-2.38**	0.23
	18.9	-39.9	4.3	175	**3.39**	-0.02	-0.72	**-2.56**	**-2.79**	-0.43

Table S1. Searchlight clusters showing mismatch sensitivity for different memory content. Coordinates (x, y, z) correspond to the voxel with the maximal t-value within each cluster extent. Statistics reflect one-sample t-tests of mismatch sensitivity (ΔRSA, near vs. far) against zero for each condition: both swap (BS), location swap (LS), and object swap (OS). Paired t-tests indicate direct comparisons between mismatch types. Post-hoc significance is indicated in **boldface** ($p < .05$, FDR-corrected across all post-hoc tests).

bioRxiv preprint doi: https://doi.org/10.1101/2025.10.15.682651; this version posted October 15, 2025. The copyright holder for this preprint (which was not certified by peer review) is the author/funder, who has granted bioRxiv a license to display the preprint in perpetuity. It is made available under aCC-BY 4.0 International license.

Seed	Cluster	x	y	z	Hemisphere	t	Size (mm^3)	Region
aHPC	1	-26.6	-13.9	-19.1	L	9.0	31536	Hippocampus
	1a	-65.6	-44.8	6.1	L	4.2		MTG, temporooccipital part
	1b	-46.1	15.4	-19.1	L	4.1		Temporal Pole
	1c	-67.2	-33.4	4.3	L	4.1		STG, posterior division
	2	28.6	-12.2	-19.1	R	5.6	8588	Hippocampus
	3	56.2	12.1	-19.1	R	5.0	5827	Temporal Pole
	3a	70.9	-35.0	7.9	R	4.2		MTG, posterior division
	4	61.1	-5.8	-26.3	R	4.9	1487	MTG, anterior division
	5	-55.9	-52.9	20.5	L	4.8	2932	Angular Gyrus
	6	61.1	-5.8	11.5	R	4.4	1259	Central Opercular Cortex
	7	-3.9	34.9	24.1	L	4.3	484	Paracingulate Gyrus
	8	9.1	-69.1	-15.5	R	4.2	1839	Lingual Gyrus
	9	62.8	-4.1	24.1	R	4.1	1026	Precentral Gyrus
	10	-41.2	36.5	34.9	L	3.9	1273	Middle Frontal Gyrus
	11	36.8	-0.9	13.3	R	3.8	551	Insular Cortex
	12	9.1	65.8	25.9	R	3.8	608	Frontal Pole
	13	-44.5	-41.5	42.1	L	3.7	789	SMG, posterior division
	14	15.6	46.2	38.5	R	3.7	632	Frontal Pole
	15	-62.4	-7.4	40.3	L	3.7	903	Postcentral Gyrus
	16	2.6	39.8	27.7	R	3.6	527	Paracingulate Gyrus
	17	10.8	-69.1	22.3	R	3.6	480	Cuneal Cortex
	18	-26.6	20.2	-22.7	L	3.5	708	Frontal Orbital Cortex
	19	-10.4	62.5	24.1	L	3.4	494	Frontal Pole

Table S2. Clusters of significant functional connectivity were identified for the anterior hippocampus (aHPC), with an FDR-corrected threshold ($p < 0.05$, $t > 2.93$), a minimum cluster size of 100 voxels, and a minimum peak-to-peak distance of 20 mm. For each cluster, the peak voxel (highest t-statistic) was extracted and labeled using the Harvard-Oxford subcortical and cortical structural atlases [115–119]. MTG, middle temporal gyrus. STG, superior temporal gyrus. SMG, supramarginal gyrus.

bioRxiv preprint doi: https://doi.org/10.1101/2025.10.15.682651; this version posted October 15, 2025. The copyright holder for this preprint (which was not certified by peer review) is the author/funder, who has granted bioRxiv a license to display the preprint in perpetuity. It is made available under aCC-BY 4.0 International license.

Seed	Cluster	x	y	z	Hemisphere	t	Size (mm^3)	Region
iHPC	1	-33.1	-23.6	-13.7	L	8.6	11540	Hippocampus
	1a	7.5	-56.1	-11.9	R	3.6		Cerebellum
	2	-55.9	-4.1	-13.7	L	5.8	15680	MTG, anterior division
	2a	-59.1	-48.0	16.9	L	4.1		SMG, posterior division
	2b	-49.4	10.5	-8.3	L	4.0		Temporal Pole
	2c	-57.5	-43.1	20.5	L	3.5		SMG, posterior division
	3	20.5	-7.4	-10.1	R	5.5	3555	Amygdala
	4	-28.2	-41.5	56.5	L	4.9	14420	Superior Parietal Lobule
	4a	-3.9	39.8	49.3	L	3.9		Superior Frontal Gyrus
	4b	-52.6	-23.6	52.9	L	3.9		Postcentral Gyrus
	4c	-3.9	49.5	47.5	L	3.9		Frontal Pole
	5	10.8	-25.2	47.5	R	4.7	12153	Precentral Gyrus
	6	-3.9	56.0	-6.5	L	4.6	560	Frontal Pole
	7	56.2	-43.1	4.3	R	4.6	983	MTG, temporooccipital part
	8	22.1	-46.4	67.3	R	4.5	13579	Superior Parietal Lobule
	8a	59.5	-17.1	49.3	R	3.8		Postcentral Gyrus
	9	-18.5	-25.2	76.3	L	4.0	860	Precentral Gyrus
	10	5.9	52.8	45.7	R	4.0	674	Frontal Pole
	11	-51.0	-41.5	56.5	L	3.7	727	SMG, posterior division
	12	15.6	-31.8	61.9	R	3.7	537	Precentral Gyrus
	13	67.6	-17.1	0.7	R	3.5	817	STG, posterior division

Table S3. Clusters of significant functional connectivity were identified for the intermediate hippocampus (iHPC), with an FDR-corrected threshold ($p < 0.05$, $t > 2.86$), a minimum cluster size of 100 voxels, and a minimum peak-to-peak distance of 20 mm. For each cluster, the peak voxel (highest t-statistic) was extracted and labeled using the Harvard-Oxford subcortical and cortical structural atlases [115–119]. MTG, middle temporal gyrus. STG, superior temporal gyrus. SMG, supramarginal gyrus.

bioRxiv preprint doi: https://doi.org/10.1101/2025.10.15.682651; this version posted October 15, 2025. The copyright holder for this preprint (which was not certified by peer review) is the author/funder, who has granted bioRxiv a license to display the preprint in perpetuity. It is made available under aCC-BY 4.0 International license.

Seed	Cluster	x	y	z	Hemisphere	t	Size (mm^3)	Region
pHPC	1	30.2	-35.0	-4.7	R	8.9	285667	Hippocampus
	1a	-13.6	-64.2	4.3	L	5.6		Intracalcarine Cortex
	1b	15.6	-2.5	-11.9	R	5.6		Cerebellum
	1c	-10.4	-64.2	0.7	L	5.5		Lingual Gyrus
	2	54.6	8.9	-17.3	R	4.6	6416	Temporal Pole
	3	-5.5	5.6	49.3	L	4.5	2281	SMA
	4	27.0	41.4	-17.3	R	4.5	3327	Frontal Pole
	5	9.1	4.0	52.9	R	4.4	4681	SMA
	5a	2.6	54.4	42.1	R	4.0		Superior Frontal Gyrus
	6	-12.0	44.6	43.9	L	4.3	1216	Frontal Pole
	7	43.2	-51.2	-51.5	R	4.1	9558	Cerebellum
	8	-33.1	-44.8	56.5	L	4.1	2813	Superior Parietal Lobule
	9	23.8	10.5	-40.7	R	3.9	1459	Temporal Pole
	10	54.6	39.8	-1.1	R	3.8	1278	Frontal Pole
	11	-41.2	43.0	-1.1	L	3.7	2542	Frontal Pole
	12	-7.1	36.5	22.3	L	3.5	1197	Cingulate Gyrus, anterior division
	13	-3.9	10.5	27.7	L	3.5	608	Cerebral White Matter
	14	53.0	-23.6	9.7	R	3.5	836	Planum Temporale
	15	-26.6	0.8	52.9	L	3.5	2752	Middle Frontal Gyrus
	16	-26.6	-52.9	69.1	L	3.4	541	Superior Parietal Lobule
	17	-8.8	-49.6	60.1	L	3.3	798	Precuneous Cortex
	18	-42.9	25.1	45.7	L	3.3	708	Middle Frontal Gyrus
	19	33.5	-46.4	-35.3	R	3.2	584	Cerebellum
	20	-38.0	-7.4	-44.3	L	2.9	1040	TFC, anterior division

Table S4. Clusters of significant functional connectivity were identified for the posterior hippocampus (pHPC), with an FDR-corrected threshold ($p < 0.05$, $t > 2.39$), a minimum cluster size of 100 voxels, and a minimum peak-to-peak distance of 20 mm. For each cluster, the peak voxel (highest t-statistic) was extracted and labeled using the Harvard-Oxford subcortical and cortical structural atlases [115–119]. SMA, supplemental motor area. TFC, temporo-fusiform cortex.

bioRxiv preprint doi: https://doi.org/10.1101/2025.10.15.682651; this version posted October 15, 2025. The copyright holder for this preprint (which was not certified by peer review) is the author/funder, who has granted bioRxiv a license to display the preprint in perpetuity. It is made available under aCC-BY 4.0 International license.

Supplementary Movies

All movies show example trials from a single participant with simultaneously recorded eye movements. The current gaze position is indicated in blue.

Movie S1. Example trial with an intact sequence.

Movie S2. Example trial with an object swap.

Movie S3. Example trial with a location swap.

Movie S4. Example trial with both an object and location swap.

Supplementary References

98. Oscar Esteban, Ross Blair, Christopher J. Markiewicz, Shoshana L. Berleant, Craig Moodie, Feilong Ma, Ayse Ilkay Isik, Asier Erramuzpe, Mathias Kent, James D. andGoncalves, Elizabeth DuPre, Kevin R. Sitek, Daniel E. P. Gomez, Daniel J. Lurie, Zhifang Ye, Russell A. Poldrack, and Krzysztof J. Gorgolewski. fmriprep. *Software*, 2018. doi: 10.5281/zenodo.852659.

99. N. J. Tustison, B. B. Avants, P. A. Cook, Y. Zheng, A. Egan, P. A. Yushkevich, and J. C. Gee. N4itk: Improved n3 bias correction. *IEEE Transactions on Medical Imaging*, 29(6):1310–1320, 2010. doi: 10.1109/TMI.2010.2046908.

100. B.B. Avants, C.L. Epstein, M. Grossman, and J.C. Gee. Symmetric diffeomorphic image registration with cross-correlation: Evaluating automated labeling of elderly and neurodegenerative brain. *Medical Image Analysis*, 12(1):26–41, 2008. doi: 10.1016/j.media.2007.06.004.

101. Y. Zhang, M. Brady, and S. Smith. Segmentation of brain MR images through a hidden markov random field model and the expectation-maximization algorithm. *IEEE Transactions on Medical Imaging*, 20(1):45–57, 2001. doi: 10.1109/42.906424.

102. Martin Reuter, Herminia Diana Rosas, and Bruce Fischl. Highly accurate inverse consistent registration: A robust approach. *NeuroImage*, 53(4):1181–1196, 2010. doi: 10.1016/j.neuroimage.2010.07.020.

103. Anders M. Dale, Bruce Fischl, and Martin I. Sereno. Cortical surface-based analysis: I. segmentation and surface reconstruction. *NeuroImage*, 9(2):179–194, 1999. doi: 10.1006/nimg.1998.0395.

104. Arno Klein, Satrajit S. Ghosh, Forrest S. Bao, Joachim Giard, Yrjö Häme, Eliezer Stavsky, Noah Lee, Brian Rossa, Martin Reuter, Elias Chaibub Neto, and Anisha Keshavan. Mindboggling morphometry of human brains. *PLOS Computational Biology*, 13(2):e1005350, 2017. doi: 10.1371/journal.pcbi.1005350.

105. R. Ciric, William H. Thompson, R. Lorenz, M. Goncalves, E. MacNicol, C. J. Markiewicz, Y. O. Halchenko, S. S. Ghosh, K. J. Gorgolewski, R. A. Poldrack, and O. Esteban. TemplateFlow: FAIR-sharing of multi-scale, multi-species brain models. *Nature Methods*, 19:1568–1571, 2022. doi: 10.1038/s41592-022-01681-2.

106. VS Fonov, AC Evans, RC McKinstry, CR Almli, and DL Collins. Unbiased nonlinear average age-appropriate brain templates from birth to adulthood. *NeuroImage*, 47, Supplement 1:S102, 2009. doi: 10.1016/S1053-8119(09)70884-5.

107. Mark Jenkinson, Peter Bannister, Michael Brady, and Stephen Smith. Improved optimization for the robust and accurate linear registration and motion correction of brain images. *NeuroImage*, 17 (2):825–841, 2002. doi: 10.1006/nimg.2002.1132.

bioRxiv preprint doi: https://doi.org/10.1101/2025.10.15.682651; this version posted October 15, 2025. The copyright holder for this preprint (which was not certified by peer review) is the author/funder, who has granted bioRxiv a license to display the preprint in perpetuity. It is made available under aCC-BY 4.0 International license.

108. Robert W. Cox and James S. Hyde. Software tools for analysis and visualization of fMRI data. *NMR in Biomedicine*, 10(4-5):171–178, 1997. doi: 10.1002/(SICI)1099-1492(199706/08)10:4/5⟨171::AID-NBM453⟩3.0.CO;2-L.

109. Douglas N Greve and Bruce Fischl. Accurate and robust brain image alignment using boundary-based registration. *NeuroImage*, 48(1):63–72, 2009. doi: 10.1016/j.neuroimage.2009.06.060.

110. Jonathan D. Power, Anish Mitra, Timothy O. Laumann, Abraham Z. Snyder, Bradley L. Schlaggar, and Steven E. Petersen. Methods to detect, characterize, and remove motion artifact in resting state fMRI. *NeuroImage*, 84(Supplement C):320–341, 2014. doi: 10.1016/j.neuroimage.2013.08.048.

111. Yashar Behzadi, Khaled Restom, Joy Liau, and Thomas T. Liu. A component based noise correction method (CompCor) for BOLD and perfusion based fMRI. *NeuroImage*, 37(1):90–101, 2007. doi: 10.1016/j.neuroimage.2007.04.042.

112. Theodore D. Satterthwaite, Mark A. Elliott, Raphael T. Gerraty, Kosha Ruparel, James Loughead, Monica E. Calkins, Simon B. Eickhoff, Hakon Hakonarson, Ruben C. Gur, Raquel E. Gur, and Daniel H. Wolf. An improved framework for confound regression and filtering for control of motion artifact in the preprocessing of resting-state functional connectivity data. *NeuroImage*, 64(1): 240–256, 2013. doi: 10.1016/j.neuroimage.2012.08.052.

113. Rémi Patriat, Richard C. Reynolds, and Rasmus M. Birn. An improved model of motion-related signal changes in fMRI. *NeuroImage*, 144, Part A:74–82, 2017. doi: 10.1016/j.neuroimage.2016.08.051.

114. C. Lanczos. Evaluation of noisy data. *Journal of the Society for Industrial and Applied Mathematics Series B Numerical Analysis*, 1(1):76–85, 1964. doi: 10.1137/0701007.

115. Jean A Frazier, Sufen Chiu, Janis L Breeze, Nikos Makris, Nicholas Lange, David N Kennedy, Martha R Herbert, Eileen K Bent, Vamsi K Koneru, Megan E Dieterich, et al. Structural brain magnetic resonance imaging of limbic and thalamic volumes in pediatric bipolar disorder. *American Journal of Psychiatry*, 162(7):1256–1265, 2005.

116. Nikos Makris, Jill M Goldstein, David Kennedy, Steven M Hodge, Verne S Caviness, Stephen V Faraone, Ming T Tsuang, and Larry J Seidman. Decreased volume of left and total anterior insular lobule in schizophrenia. *Schizophrenia Research*, 83(2-3):155–171, 2006. doi: 10.1016/j.schres.2005.11.020.

117. Rahul S Desikan, Florent Ségonne, Bruce Fischl, Brian T Quinn, Bradford C Dickerson, Deborah Blacker, Randy L Buckner, Anders M Dale, R Paul Maguire, Bradley T Hyman, et al. An automated labeling system for subdividing the human cerebral cortex on MRI scans into gyral based regions of interest. *NeuroImage*, 31(3):968–980, 2006.

118. Jill M Goldstein, Larry J Seidman, Nikos Makris, Todd Ahern, Liam M O'Brien, Verne S Caviness Jr, David N Kennedy, Stephen V Faraone, and Ming T Tsuang. Hypothalamic abnormalities in schizophrenia: sex effects and genetic vulnerability. *Biological Psychiatry*, 61(8): 935–945, 2007.

119. Rastko Ciric, William H Thompson, Romy Lorenz, Mathias Goncalves, Eilidh E MacNicol, Christopher J Markiewicz, Yaroslav O Halchenko, Satrajit S Ghosh, Krzysztof J Gorgolewski, Russell A Poldrack, et al. Templateflow: Fair-sharing of multi-scale, multi-species brain models. *Nature Methods*, 19(12):1568–1571, 2022.
